# Fire on the Hearth

**Published:** 1869-02

**Authors:** 


### FIRE ON THE HEARTH.

At the close of six thousand years no plan has ever been devised for warming single rooms healthfully and pleasantly equal to the old-fashioned wood fires in generously broad fire-places. The next thing to it, with two very great advantages, is the LOW-DOWN GRATE, made by T. S. Dixon & Sons, of Philadelphia, whose agent is Geo. E. Woodward, 191 Broadway, New York City. The advantages are less trouble, less expense and greater heat. These grates have the fuel placed on the hearth, on a level with the floor. They will burn wood or peat or soft coal, or even the hardest anthracite coal, without the necessity of a "blower" to kindle. The fire needs no replenishing for half a day at a time; needs no poker, and presents all the time a broad bed of glowing coals, which can be extinguished in a few minutes without water, without dust or any other discomfort, while the ventilation of the room is thorough, and the heat is soft and balmy and sweet.

